# Clinical Investigation of the Posterior scleral contraction to Treat Macular Traction Maculopathy in Highly Myopic Eyes

**DOI:** 10.1038/srep43256

**Published:** 2017-02-21

**Authors:** An-Peng Pan, Ting Wan, Shuang-Qian Zhu, Liang Dong, An-Quan Xue

**Affiliations:** 1 The Eye Hospital of Wenzhou Medical University, 270 Xueyuan West Road, Wenzhou 325000, Zhejiang, P.R. China

## Abstract

Myopic traction maculopathy (MTM) can cause vision disabilities in highly myopic eyes. This retrospective case series investigated the clinical outcomes of posterior scleral contraction (PSC) using genipin-cross-linked sclera as the material to treat MTM in highly myopic eyes. In total, 32 eyes from 29 highly myopic patients who underwent PSC for MTM were recruited. The changes in best-corrected visual acuity (BCVA) and axial length were evaluated, macular reattachment and macular hole (MH) closure was assessed by optical coherence tomography, and complications were evaluated. At the final follow-up, the retina was completely reattached in 25 eyes (78.1%), essentially reattached in 4 eyes (12.5%), and partially reattached in 3 eyes (9.4%). The logMAR BCVA improved significantly from 1.18 ± 0.45 preoperatively to 0.87 ± 0.45 postoperatively (P < 0.001). The 32 eyes were further divided into the MH group (16 eyes) and the non-MH group (16 eyes) for comparison. The MH was closed in 9 eyes (56.3%). The retinal reattachment rate was 75.0% in the MH group and 81.25% in the non-MH group, and the logMAR BCVA improved significantly in both groups. The PSC using genipin-cross-linked sclera as the material can effectively treat MTM in highly myopic eyes, and significant visual improvement can be achieved with minimal complications.

The prevalence of high myopia is increasing, and this disease has become a major cause of blindness in the Asian population^1^,^2^,^3^,^4^,^5^,^6^. Myopic traction maculopathy (MTM) is one of the severe complications that can potentially cause visual disabilities in highly myopic eyes^7^,^8^,^9^,^10^. Macular hole retinal detachment (MHRD) is considered to be the final stage of MTM and requires surgical treatment to preserve visual function. Although myopic foveoschisis (MF) is considered to be the initial stage of MTM, it tends to be stable for long periods of time^11^,^12^, and the presence of foveal detachment (FD) associated with MF often warrants treatment to prevent progression of the disease towards a stage of poorer visual and anatomic prognosis^10^,^13^,^14^.

Currently, surgical treatment for MTM in highly myopic eyes includes pars plana vitrectomy (PPV) with gas tamponade with or without internal limiting membrane (ILM) peeling, PPV with silicone oil tamponade, macular buckle, and posterior scleral reinforcement (PSR) alone or combined with vitrectomy^15^,^16^,^17^,^18^,^19^,^20^,^21^,^22^,^23^,^24^. Among the various surgical techniques used to treat MTM, the most common procedure used currently is PPV with gas tamponade with ILM peeling. Vitrectomy with ILM peeling can release vitreous traction and abnormally rigid ILM to treat MTM effectively, but the retinal reattachment rate for MHRD, which is considered to be the final stage of MTM and one of the most challenging complications of high myopia, is variable, ranging from 63.2% to 86% with an even lower hole closure rate^25^,^26^,^27^,^28^. Meanwhile, visual improvement was hard to achieve despite anatomical and surgical success in eyes with MHRD^21^,^25^,^29^, and reoperations are still needed in certain cases^27^,^30^. The reason for poor functional and anatomical outcomes may be that the vitrectomy alone does not address the pulling effect exerted by the posterior staphyloma or prevent the stretching of the posterior sclera. On the other hand, retinal thinning and chorioretinal atrophy continue after vitrectomy. On the contrary, the PSR or macular buckle as a surgery to prevent the stretching of the posterior sclera can relieve the traction on the retina from the outer sclera and seems to be an acceptable approach that has been proven to be effective to treat MTM^22^,^31^,^32^. In recent years, clinical applications of the cross-linking technology and biological membrane materials have increased. The cross-linked biological membrane exhibits increased toughness and mechanical strength, enhanced elastic modulus and good histocompatibility^33^,^34^,^35^,^36^,^37^. We cross-linked the donor sclera as the surgical material to treat MTM, and the standard PSR was modified. More importantly, the axial length was shortened intentionally by 1/10 of the preoperative axial length, and the vitreous volume was reduced such that the surgery was named a posterior scleral contraction (PSC).

In the present study, we retrospectively reviewed the clinical outcomes of PSC using genipin-cross-linked sclera as the material to treat MTM in highly myopic eyes. The efficacy and safety of PSC were evaluated for more than 6 months postoperatively.

## Methods

The inclusion criteria included a spherical equivalent ≥−6.00 Diopters (D) and being diagnosed with high myopia. The preoperative optical coherence tomography (OCT) was reviewed to confirm the presence of MTM, and subjects received the PSC surgery with genipin-cross-linked sclera as the material. Subjects with a history of ocular trauma or surgery (other than cataract surgery), choroidal neovascularization and severe cataracts were excluded from the investigation. Overall, 32 eyes from 29 consecutive patients with MTM were included in this retrospective study, and the PSC surgeries were performed from January 2013 to April 2015 at the Eye Hospital of Wenzhou Medical University. The pre- and postoperative data, including the best-corrected visual acuity (BCVA) by logMAR, axial length, fundus photo, intraocular pressure (IOP) and OCT images, were collected by reviewing the medical records of all subjects. The AL was measured via an optical biometer (IOL Master, Zeiss, Germany), and high-resolution OCT (Carl Zeiss Meditec Inc., Dublin, California, USA) images were used to evaluate the retinal reattachment. This study followed the tenets of the Declaration of Helsinki and was approved by the Ethics Committee of the Eye Hospital of Wenzhou Medical University. Signed informed consent that allowed use of the data for research was obtained from each patient.

### Preparation of the sclera

**Figure 1:**
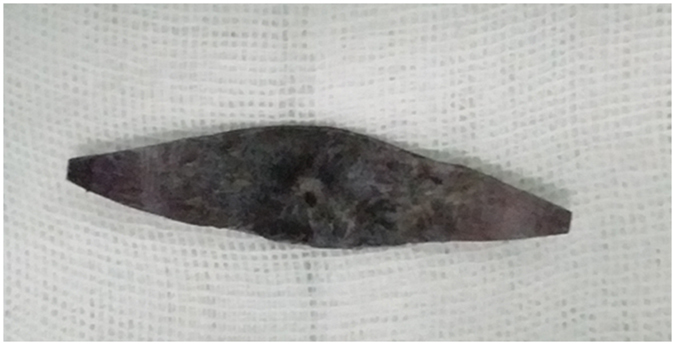
The genipin-cross-linked sclera used in posterior scleral contraction (PSC) surgery. The donor sclera was cut into spindle strips.

Human sclera was obtained from a local eye bank after the cornea had been removed for transplantation. The donor sclera was cleaned and soaked in a mixed solution of 0.05%~0.5% genipin (Wako, Japan) and 20%~40% alcohol at 20~30 °C for 3~5 weeks. Then, the sclera was sterilized by 3% povidone iodine solution for 12~24 hours, and 75% alcohol was used for deiodination. Bacteriological tests were conducted on all the sclerae to confirm sterility. Prior to use, the genipin-cross-linked sclerae were kept in an airtight container full of 75% alcohol. During the surgery, the donor sclera was cut into spindle strips (Fig. 1). The designed length of the scleral strip was 1.5 times the axial length, the width was 0.4 times the axial length in the middle and 3~5 mm at both ends, and the sclera strip was rinsed with normal saline before use.

### Surgical procedure

**Figure 2:**
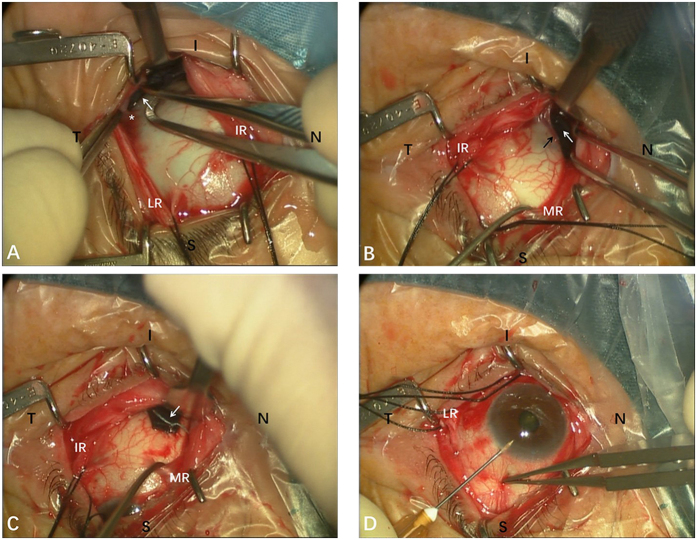
Intraoperative photographs of the posterior scleral contraction (PSC) surgery in one case (Left eye). (**A**) With the assistance of the traction sutures, the sclera strip (white arrow) was passed underneath the inferior oblique, next to the insertion point of the inferior oblique (asterisk). (**B**) The sclera strip (white arrow) was passed underneath the inferior rectus with caution, efforts were made to protect vortex vein (black arrow) from damage. (**C**) The inferior nasal end of sclera strip (white arrow) was fixed to the pre-equatorial sclera, 2 mm behind and outside the insertion point of the medial rectus (MR) muscle. (**D**) A 25-gauge syringe needle (1 mL) was inserted into the superior anterior chamber to release the aqueous humor. S: superior; I: inferior; N: nasal; T: temporal; LR: lateral rectus; IR: inferior rectus; MR: medial rectus.

**Figure 3:**
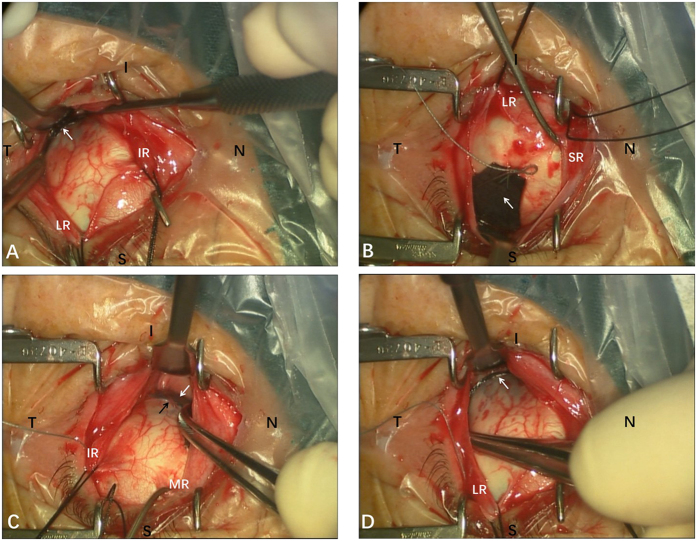
Intraoperative photographs of the posterior scleral contraction (PSC) surgery, the same case as in Fig. 2. (**A**) Ensured that the middle part of the sclera strip (white arrow) was placed at the posterior pole. (**B**) The superior temporal end of sclera strip (white arrow) was fixed to the pre-equatorial sclera, 2 mm behind and outside the insertion point of the superior rectus (SR) muscle. (**C**) The scleral strip (white arrow) was checked to assure that it was correctly positioned without damaging the vortex vein (black arrow). (**D**) Ensured that the sclera strip (white arrow) was stretched into a U shape to surround the posterior pole. S: superior; I: inferior; N: nasal; T: temporal; LR: lateral rectus; IR: inferior rectus; MR: medial rectus; SR: superior rectus.

The surgical technique for posterior scleral reinforcement was published previously^31^,^38^. The PSC was a modified version of PSR, and more importantly, the axial length was shortened and vitreous volume was reduced. The Intraoperative photographs of the PSC surgery in one case were showed in Figs 2 and 3. Schematic drawings which depicted the relationships between the sclera strip and the landmarks of the posterior globe has been published previously^38^. The surgical procedure was as follows. PSC was performed under general anesthesia. A 210° peritomy of the conjunctiva was performed along the inferior temporal axis of the limbus. A radial cut was made at each end of the peritomy to expose the inferior and lateral rectus muscles, and traction sutures were prepared for these muscles. With the assistance of the traction sutures and a muscle hook that lifted up the complete inferior oblique, the sclera strip was sequentially passed underneath the inferior oblique (Fig. 2A), lateral rectus and inferior rectus muscles. During this process, efforts were made to protect the optic nerve and vortex veins from damage (Fig. 2B). Firstly, the middle part of the sclera strip was placed at the posterior pole, and the inferior nasal end was fixed to the pre-equatorial sclera, 2 mm behind and outside the insertion point of the medial rectus muscle (Fig. 2C). Secondly, before suturing the superior temporal end, approximately 0.05~0.2 mL aqueous humor was released using a 25-gauge syringe needle (1 mL) inserted into the superior anterior chamber (Fig. 2D). Thirdly, after making sure that the middle part of the sclera strip was placed at the posterior pole (Fig. 3A), the sclera strip was tightened by pulling, and the superior temporal end was fixed to the pre-equatorial sclera, 2 mm behind and outside the insertion point of the superior rectus muscle (Fig. 3B). The expected shortening of the axial length was 1/10 of the axial length. The scleral strip was stretched into a U shape to surround the posterior pole. Before the end of the procedure, the scleral strip was checked to assure that it was correctly positioned and oriented (Fig. 3C and D). The posterior pole was inpsected with indirect ophthalmoscopy to ensure that the optic nerve head and major blood vessels were normal and that the macular was slightly bulged. Finally, the conjunctival incision was closed using 8–0 absorbable sutures. Postoperatively, dexamethasone (0.1%) and antibiotic eye drops were recommended for use four times daily for 3 weeks.

### Retinal reattachment assessed by OCT

The macular 12 azimuth scan OCT images were reviewed for all subjects. The retinal detachment area (the azimuth scan of maximal detachment was used) was compared before and after surgery (at each follow-up), and the retina reattachment was divided into four categories base on the following crieteria: completely reattached, the retinal detachment completely disappeared and the retina was successful reattached; essentially reattached, the retinal detachment area was reduced by more than 80%; partially reattached, the retinal detachment area was reduced by 40%~79%; and not reattached, the retinal detachment area was reduced by less than 39%.

### Statistical analysis

All statistical analyses were performed using SPSS 16.0 (SPSS Inc., Chicago, Illinois, USA). All continuous variables are expressed as the means ± standard deviations. Preoperative and postoperative parameters, including logMAR BCVA and AL values, were compared by paired t-test to assess if changes were significant. A p-value of less than 0.05 was considered significant.

## Results

The patient demographics are described in Table 1. Twenty-six of the patients were women (29 eyes), and 3 were men (3 eyes). The mean age was 55.97 ± 12.31 years (range 33–80 years). Based on the OCT findings, 12 eyes were diagnosed with MHRD and 4 eyes had lamellar holes. Among all the eyes, 18 eyes had foveal retinal detachment and foveal retinoschisis, 6 eyes had only foveal retinal detachment, and 8 eyes had foveal retinal detachment, foveal retinoschisis and epiretinal membrane. The mean follow-up time was 15.4 ± 7.6 months.

### Rate of retinal reattachment, macular hole closure, visual acuity and axial length

**Figure 4:**
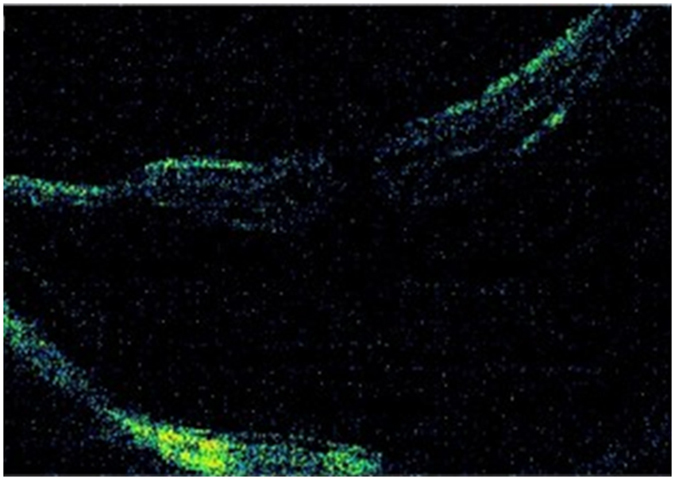
The patient was 59 years old, female, with a logMAR BCVA of 1.30, and the axial length was 29.99 mm. The preoperative optical coherence tomography (OCT) image of the left eye revealed a significant macular hole retinal detachment (MHRD).

**Figure 5:**
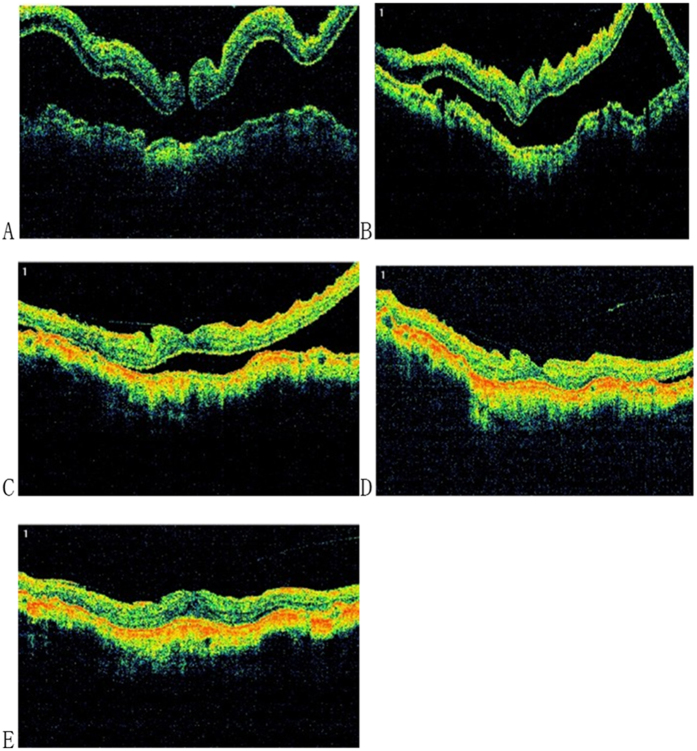
The patient’s left eye received posterior scleral contraction (PSC) in June 2013, the genipin-cross-linked sclera was spindle shaped, and the length of the sclera strip was 45 mm with a width of 12 mm in the middle. (**A**) One week after the surgery, significant retinal folds were noted, and the macular hole was still present. The axial length (AL) was 27.09 mm with a reduction of 2.90 mm compared to the preoperative AL (29.99 mm), and the LogMAR BCVA was 1.30. (**B**) One month after the surgery, the retinal detachment area was reduced, the AL was 27.47 mm, and the logMAR BCVA was 1.0. (**C**) Six months after the surgery, the retinal detachment area continued to reduce, retinal folds were relieved, the AL was 27.78 mm, and the logMAR BCVA was 1.0. (**D**) Twelve months after the surgery, the retina was essentially reattached, the AL was 27.83 mm, and the logMAR BCVA was 1.0. (**D**) Twenty-four months after surgery, the retina was completely reattached, the AL was 27.78 mm with a reduction of 2.21 mm compared to the preoperative AL (29.99 mm), and the logMAR BCVA was 0.7.

**Figure 6:**
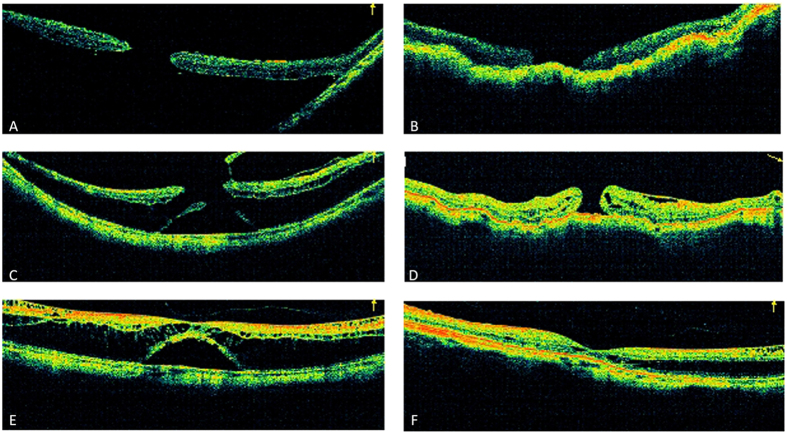
(**A**) For eye No. 8, preoperatively, OCT images showed a huge macular hole and associated retinal detachment. The logMAR best-corrected visual acuity was 2.0. The axial length was 30.0 mm. (**B**) After posterior scleral contraction (PSC), there was a complete retinal reattachment, but the macular hole was not closed. The logMAR best-corrected visual acuity was 1.4. The axial length was 26.63 mm. (**C**) For eye No. 26, preoperatively, OCT images showed a macular hole and associated retinal detachment, and the epiretinal membrane was present as well. The logMAR best-corrected visual acuity was 0.52. The axial length was 30.49 mm. (**D**) After PSC, the macular hole was not closed, and the retinal detachment area was reduced by more than 80% but not completely. The retinal detachment was still present around the macular hole edge, which was defined as essential reattachment. The logMAR best-corrected visual acuity was 0.4. The axial length was 27.88 mm. (**E**) For eye No. 31, preoperatively, OCT images showed foveal detachment and myopic foveoschisis. The logMAR best-corrected visual acuity was 0.52. The axial length was 27.98 mm. (**F**) After PSR, the retinal detachment area was reduced by 40%~79%, and retinal detachment was still present, which was defined as partial reattachment. The logMAR best-corrected visual acuity was 0.22. The axial length was 26.87 mm.

At the final follow-up, the retina was completely reattached in 25 eyes (78.1%), essentially reattached in 4 eyes (12.5%), and partially reattached in 3 eyes (9.4%). The logMAR BCVA improved significantly at final follow-up from 1.18 ± 0.45 preoperatively to 0.87 ± 0.45 postoperatively (P < 0.001). The logMAR BCVA was improved in 26 eyes (81.25%) and unchanged in 6 eyes (18.75%). The axial length at the finial follow-up was 27.60 ± 2.08 mm when compared to the preoperative axial length (29.76 ± 2.23 mm), where the axial length reduction was 2.16 ± 0.62 mm, which is slightly regressed compared to the axial length reduction at the 1-week follow-up (2.73 ± 0.64 mm). All 32 eyes were further divided into the MH group (16 eyes) and the non-MH group (16 eyes) for comparison, and there were no significant differences between the two groups in age, follow-up, preoperative AL or preoperative logMAR BCVA. In the MH group, the MH was closed in 9 eyes (56.3%). The features of the macular hole and the surgical outcomes are presented in Table 2. The retinal reattachment rate was 75.0% (12/16) in the MH group and 81.25% (13/16) in the non-MH group, and the logMAR BCVA improved significantly at the final follow-up from 1.12 ± 0.43 preoperatively to 0.81 ± 0.43 postoperatively (P < 0.001) in the MH group and from 1.25 ± 0.47 to 0.92 ± 0.48 (P < 0.001) in the non-MH group. There was no significant difference in the improvement in logMAR BCVA, pre- or postoperatively between the MH group and the non-MH group (0.31 ± 0.22 vs 0.32 ± 0.29, P > 0.05). The clinical information for a typical eye with MHRD (Eye No. 6) who had received PSC is presented in Figs 4 and 5. Figure 6 shows the pre- and postsurgery OCT images of 3 eyes.

### Complications

The early postoperative OCT showed iatrogenic retinal folds (Fig. 5A), which were more significant in the eyes with MF, and the retinal folds were gradually flattened, accompanied by the reattaching of the retina. No obvious macular atrophy was noted during follow-up by carefully comparing the pre- and postoperative fundus photos. There were no severe complications such as vitreous hemorrhage, persistent elevated intraocular pressure, rejection or infection during the follow-up period.

## Discussion

For the high myopia patient, axial elongation continues as the patient ages^39^, and in patients with MTM, the mismatch between the retina and the outer choroid-sclera complex associated with posterior staphyloma and axial elongation is a major cause of the traction over the macula^40^,^41^. The mismatch may also be the reason for the failure of the PPV to reattach the retina and close the MH in refractory cases. The PSC used in this study, aimed at one of the main causative factors of MTM, was an intervention to match the outer choroid-sclera complex with the inner retina to achieve the therapeutic effect. In this study, the retina was completely reattached in 25 eyes (78.1%), essentially reattached in 4 eyes (12.5%), and partially reattached in 3 eyes (9.4%), which indicated that the PSC was effective at reattaching the retina in eyes with MTM by relieving the traction over the macular. The PSC was not only a way to strengthen the weak posterior sclera but also a way to contract the expanded posterior sclera. The choroid/retina was also contracted simultaneously, and this can be proved by the retinal folds shown in the early postoperative OCT images. We observed that the retinal folds were gradually flattened, and the retinal detachment was gradually reattached as well during the follow-up, which was consistent with our previous study^31^ and was considered a rematch of the retina to the outer choroid-sclera complex after the sclera expansion remitted.

Several studies^22^,^31^,^41^,^42^,^43^ that tried to deal with the mismatch between the retina and the outer choroid-sclera complex by other buckling techniques have been reported. Ohba *et al*.^41^ compared the outcomes of episcleral macular buckling with PPV for MHRD in highly myopic eyes, and they found that the episcleral macular buckling with a macular plomb achieved a retinal reattachment rate of 93.3%, which was better than PPV, after primary surgery. The higher anatomical success rate in Ohba’s study compared to our study (93.3% in Ohba’s study vs 75.0% in the MH group) can be explained by (1) the longer follow-up (a mean of 52.8 months in Ohba’s study vs a mean of 15.4 months in our study), (2) the different surgical materials and surgical procedures, and (3) the different ways to assess retinal reattachment (three-mirror contact lens biomicroscopy in Ohba’s study vs OCT scans in our study). Parolini *et al*.^22^ investigated the efficacy of an L-shaped macular buckle to treat MTM in highly myopic eyes, and they found that the L-shaped macular buckle alone can achieve 100% retinal reattachment and 60% MH closure in MTM. The MH closure rate was comparable to our study. The different surgical materials, surgical procedures and patient characteristics are the likely reason for the discrepancy of retinal reattachment rates between Parolini’s study and our study.

The amount of sclera contraction can be reflected by the shortening of the AL. During the surgery, the antedisplacement of the superior temporal end of the sclera strip determined the amount of AL shortening, and the expected shortening of the AL was 1/10 of the AL. The anterior chamber puncture can soften the eyeball to ease the shortening of the AL and avoid the postoperative spike of the IOP. In this study, the axial length reduction was 2.73 ± 0.64 mm at one week after surgery, which indicated that the expected amount of sclera contraction was successfully achieved. If the sclera shortening was insufficient, the sclera expansion cannot be remitted sufficiently; if the sclera shortening was excessive, the retinal folds can be severe. Both situations were antagonistic to retina reattachment. In this study, 3 eyes had partially reattached more than one year postoperatively, and this partial reattachment may be related to the insufficient sclera shortening and the reduction of the AL to 1.11 mm in one eye.

The maintenance of posterior sclera contraction was hugely related to the stability of the sclera strip *in vivo*. Degradation of the implanted human sclera has been reported^38^, and this degradation can cause loss of the therapeutic effect long-term. The cross-linked biological membrane exhibited increased toughness and mechanical strength, enhanced elastic modulus and good histocompatibility^33^,^34^,^35^,^36^,^37^. Studies^35^,^36^,^37^ have reported the safety of genipin as a chemical to cross-link the sclera. After cross-linking by genipin, the human sclera exhibited an improved ability to resist deformation. Zhu’s study^31^ found a slow increase in the AL during the initial few months after performing the posterior scleral reinforcement using genipin-cross-linked sclera, but AL was stabilized at 6 months after the operation. In our study, the reduction of the AL was 2.73 ± 0.64 mm one week after surgery and was 2.16 ± 0.62 mm at the final follow-up, which indicated that there was a regression of the AL reduction initially, but the AL was then maintained well enough to provide sufficient therapeutic effect with no re-detachment noticed at the final follow-up. On the other hand, the genipin-cross-linked sclera used in this study was dark blue in color (Fig. 1), which increased the visibility and was easy to recognize during the surgery.

The closure rate for the MH was thought to be important for determining the visual outcome after surgery^28^. In the literature, the MH closure rates after PPV varied, ranging from 31% to 63%^16^,^25^,^28^,^44^. The exact pathogenesis of MHRD in highly myopic eyes remains unclear. Anteroposterior vitreous traction^45^,^46^ and tangential vitreous traction^10^,^47^ were thought to be the causative factors for macular hole formation, but in highly myopic eyes, the extensive chorioretinal atrophy and posterior staphyloma can play important roles in developing MHRD. Studies^22^,^31^,^43^,^48^,^49^ have shown the effectiveness of various methods (PSR, macular buckle, or posterior episcleral buckling) that addressed posterior staphyloma to treat MTM. Ripandelli *et al*.^43^ reported that better visual and anatomic outcomes were achieved by posterior episcleral buckling compared with vitrectomy for MHRD in highly myopic eyes, and they believed that the scleral buckle can reduce the posterior staphyloma and release the tangential traction over the macula to facilitate the closure of the macular hole. In the present study, by means of PSC, the AL was shortened and the posterior sclera was contracted, which then may relieve the traction over the macula. As a result, the MH was closed in 9 eyes (56.3%), and the closure rate was comparable with those by means of PPV reported in the literature. Our study also found that 3 of 4 eyes (75%) with a lamellar hole were closed, 4 of 5 eyes (80%) with an MH size smaller than 500 μm were closed, and 2 of 7 eyes (28.6%) with an MH size larger than 500 μm were closed. These results indicated that the closure rate of the macular hole after PSC was hugely affected by the preoperative features of the hole, and a lamellar hole or small macular hole (<500 μm) had higher closure rates when compared to a large macular hole (>500 μm). For the eyes with a lamellar hole or small macular hole, PSC alone may be sufficient to achieve anatomic success, but for the eyes with a large macular hole (>500 μm), macular hole closure may be hard to achieve without releasing the inner traction from the vitreous. Combined surgery, PSC combined with PPV, which dealt with both intraocular and outer ocular traction, may be an effective approach to improving anatomic success.

The logMAR BCVA was significantly improved (P < 0.01) after the PSC, which was related to remission of the retina expansion and maintenance of the structure of the posterior pole. Studies^21^,^25^,^29^ have shown that visual improvement was hard to achieve, despite the anatomical and surgical success, by PPV with gas tamponade with or without ILM peeling in highly myopic eyes with MHRD. Taniuchi *et al*.^21^ evaluated the efficacy of PPV with or without ILM peeling for each stage of MTM, and improvement in vision was found in the MF and FD groups but was not found in the MH group. They believe that MHRD is the late stage of MTM, and once progressed to the MH stage, it was difficult to achieve a significant improvement in vision. MHRD was believed to be a decompensation of the retina volume to the enlarged posterior wall caused by various factors. Theoretically, in eyes with posterior staphyloma and axial elongation, PPV combined with gas tamponade may force the detached and relatively insufficient retina against the enlarged outer choroid-sclera complex, and the retina around the macula may be centrifugally stretched, which may further deteriorate the retinal function if decompensation already existed. On the contrary, the PSC can modify the posterior pole configuration by means of sclera contraction, which can reattach the retina and correct the mismatch between the retina and the choroid-sclera complex without stretching the macula. In our study, vision was improved in the MH group, and there was no significant difference in the improvement in logMAR BCVA between the MH group and non-MH groups, which indicated that the PSC was effective in treating MHRD in highly myopic eyes by contracting the posterior sclera and matching the retina with the outer choroid-sclera complex.

The limitations of this study include the relatively low number of eyes. The study may also be limited by its retrospective design and short follow-up period. Moreover, several factors that may affect the visual outcome and anatomic success were not assessed in this study, including the duration of the visual symptoms and the height of retinal detachment. Therefore, prospective studies with longer follow-up, large sample size and complete information are necessary in the future to confirm the findings of this study.

This study analyzed the changes in logMAR BCVA and AL before and after PSC surgery, along with the rate of retinal reattachment and macular hole closure to investigate the clinical outcomes of PSC using genipin-cross-linked sclera as the material to treat MTM in highly myopic eyes. The results showed that the PSC can effectively treat MTM in highly myopic eyes with minimal complications, and it can also provide good anatomic results, stable retinal reattachment, satisfactory macular hole closure rate and improved visual acuity. More importantly, visual improvement can be achieved in eyes with MHRD. In conclusion, PSC can be a valuable surgical option to treat MTM.

## Additional Information

**How to cite this article:** Pan, A.-P. *et al*. Clinical Investigation of the Posterior scleral contraction to Treat Macular Traction Maculopathy in Highly Myopic Eyes. *Sci. Rep.* **7**, 43256; doi: 10.1038/srep43256 (2017).

**Publisher's note:** Springer Nature remains neutral with regard to jurisdictional claims in published maps and institutional affiliations.

## Figures and Tables

**Table 1:** Outcomes of posterior scleral contraction (PSC) to treat myopic traction maculopathy (MTM) in highly myopic eyes at the last follow-up.

Eye No.	Gender	Age (years)	Before operation		MH	OCT	After operation		Reduction of AL (mm)	Retina reattachment	BCVA Change	Follow-up (months)
			LogMAR BCVA	AL (mm)			LogMAR BCVA	AL (mm)				
1	F	65	1.3	30.52	Yes	FD, MF	0.7	28.05	2.47	Completely	0.6	18
2	M	62	0.8	32.08	Yes, lamellar hole	FD, MF	0.4	29.34	2.74	Completely	0.4	24
3	F	48	1.3	31.43	No	FD, MF	0.7	29.45	1.98	Completely	0.6	12
4	F	74	0.52	29.29	No	FD, MF, ERM	0.52	26.83	2.46	Completely	0	12
5	F	59	0.7	32.63	Yes, lamellar hole	FD, MF	0.4	29.73	2.9	Completely	0.3	34
6	F	59	1.3	29.99	Yes	FD	0.7	27.78	2.21	Completely	0.6	24
7	F	55	0.82	31.13	Yes	FD, MF, ERM	0.8	29.64	1.49	Completely	0.03	9
8	F	64	2	30	Yes	FD	1.4	26.63	3.37	Completely	0.6	30
9	F	37	1.4	27.95	No	FD, MF	0.8	26.1	1.85	Completely	0.6	24
10	F	37	1.4	29.14	Yes, lamellar hole	FD, MF, ERM	1.3	27.88	1.26	Completely	0.1	24
11	F	69	0.7	33.95	No	FD, MF	0.52	31.57	2.38	Completely	0.18	8
12	F	59	1.3	26.9	No	FD, MF, ERM	0.8	23.98	2.92	Completely	0.5	22
13	F	39	1	27.99	No	FD, MF	0.7	26.12	1.87	Completely	0.3	18
14	F	39	1	28.44	No	FD, MF	0.7	26.04	2.4	Completely	0.3	18
15	F	52	1.3	29.63	Yes, lamellar hole	FD, MF	0.7	27.39	2.24	Completely	0.6	12
16	F	33	1	32.01	Yes	FD	0.8	30.45	1.56	Completely	0.2	10
17	F	58	1.3	28.48	No	FD, MF	1.3	26.22	2.26	Completely	0	15
18	F	51	1	34.56	Yes	FD, MF	0.7	32.74	1.82	Completely	0.3	11
19	F	57	1.3	25.44	No	FD, MF, ERM	0.7	23.91	1.53	Completely	0.6	7
20	M	79	0.8	27.6	Yes	FD, MF, ERM	0.52	25.63	1.97	Completely	0.28	7
21	F	65	1.3	28.73	Yes	FD	0.8	26.33	2.4	Completely	0.5	8
22	F	50	2	33.85	No	FD, MF	1	30.52	3.33	Completely	1	6
23	F	63	2	26.17	No	FD, MF	1.7	25.07	1.1	Completely	0.3	13
24	M	43	1	28.9	No	FD	0.52	27.8	1.1	Completely	0.48	6
25	F	52	1.3	31.43	No	FD, MF	1.3	29.27	2.16	Completely	0	8
26	F	59	0.7	27.86	Yes	FD, MF	0.52	26.47	1.39	Essentially	0.18	30
27	F	62	0.52	30.49	Yes	FD, MF, ERM	0.4	27.88	2.61	Essentially	0.12	12
28	F	59	0.92	30.48	Yes	FD, MF	0.8	27.73	2.75	Essentially	0.12	12
29	F	58	1.3	29.05	No	FD, MF, ERM	1.3	26.76	2.29	Essentially	0	15
30	F	80	2	30.48	No	FD	2	27.86	2.62	Partially	0	18
31	F	36	0.52	27.98	No	FD, MF	0.22	26.87	1.11	Partially	0.3	15
32	F	68	2	27.73	Yes	FD, MF	2	25.3	2.43	Partially	0	12

AL: axial length; BCVA: best-corrected visual acuity; OCT: optical coherence tomography; ERM: epiretinal membrane; FD: foveal detachment; MF: myopic foveoschisis; MH: macular hole; Y: yes; N: no.

**Table 2:** The features and outcomes of the macular hole in 16 eyes with macular hole retinal detachment (MHRD) in highly myopic eyes.

Eye No.	Before operation		MH closure
	AL (mm)	MH feature	
1	30.52	<500 um	closed
2	32.08	lamellar hole	closed
5	32.63	lamellar hole	closed
6	29.99	<500 um	closed
7	31.13	>500 um	closed
8	30	>1000 um	not closed
10	29.14	lamellar hole	not closed
15	29.63	lamellar hole	closed
16	32.01	<300 um	closed
18	34.56	<300 um	closed
20	27.6	>500 um	closed
21	28.73	>500 um	not closed
26	27.86	>500 um	not closed
27	30.49	>500 um	not closed
28	30.48	>500 um	not closed
32	27.73	<500 um	not closed

## References

[b1] Buch H., Vinding T. & Nielsen N. V. Prevalence and causes of visual impairment according to World Health Organization and United States criteria in an aged, urban Scandinavian population: the Copenhagen City Eye Study. Ophthalmology 108, 2347–2357 (2001). 11733284 10.1016/s0161-6420(01)00823-5

[b2] Iwase A. . Prevalence and causes of low vision and blindness in a Japanese adult population: the Tajimi Study. Ophthalmology 113, 1354–1362, doi: 10.1016/j.ophtha.2006.04.022 (2006). 16877074

[b3] Wang Y., Xu L. & Jonas J. B. Prevalence and causes of visual field loss as determined by frequency doubling perimetry in urban and rural adult Chinese. American journal of ophthalmology 141, 1078–1086, doi: 10.1016/j.ajo.2006.01.023 (2006). 16765676

[b4] Yamada M. . Prevalence of visual impairment in the adult Japanese population by cause and severity and future projections. Ophthalmic epidemiology 17, 50–57, doi: 10.3109/09286580903450346 (2010). 20100100

[b5] Wong T. Y., Ferreira A., Hughes R., Carter G. & Mitchell P. Epidemiology and disease burden of pathologic myopia and myopic choroidal neovascularization: an evidence-based systematic review. Am J Ophthalmol 157, 9–25 e12, doi: 10.1016/j.ajo.2013.08.010 (2014). 24099276

[b6] Tang Y. . Prevalence and Causes of Visual Impairment in a Chinese Adult Population: The Taizhou Eye Study. Ophthalmology 122, 1480–1488, doi: 10.1016/j.ophtha.2015.03.022 (2015). 25986897

[b7] Liu H. H. . Prevalence and progression of myopic retinopathy in Chinese adults: the Beijing Eye Study. Ophthalmology 117, 1763–1768, doi: 10.1016/j.ophtha.2010.01.020 (2010). 20447693

[b8] Panozzo G. & Mercanti A. Optical coherence tomography findings in myopic traction maculopathy. Archives of ophthalmology 122, 1455–1460, doi: 10.1001/archopht.122.10.1455 (2004). 15477456

[b9] Takano M. & Kishi S. Foveal retinoschisis and retinal detachment in severely myopic eyes with posterior staphyloma. American journal of ophthalmology 128, 472–476 (1999). 10577588 10.1016/s0002-9394(99)00186-5

[b10] Ishida S., Yamazaki K., Shinoda K., Kawashima S. & Oguchi Y. Macular hole retinal detachment in highly myopic eyes: ultrastructure of surgically removed epiretinal membrane and clinicopathologic correlation. Retina 20, 176–183 (2000). 10783951

[b11] Benhamou N., Massin P., Haouchine B., Erginay A. & Gaudric A. Macular retinoschisis in highly myopic eyes. American journal of ophthalmology 133, 794–800 (2002). 12036671 10.1016/s0002-9394(02)01394-6

[b12] Shimada N. . Natural course of macular retinoschisis in highly myopic eyes without macular hole or retinal detachment. American journal of ophthalmology 142, 497–500, doi: 10.1016/j.ajo.2006.03.048 (2006). 16935601

[b13] Baba T. . Prevalence and characteristics of foveal retinal detachment without macular hole in high myopia. American journal of ophthalmology 135, 338–342 (2003). 12614751 10.1016/s0002-9394(02)01937-2

[b14] Kobayashi H. & Kishi S. Vitreous surgery for highly myopic eyes with foveal detachment and retinoschisis. Ophthalmology 110, 1702–1707, doi: 10.1016/S0161-6420(03)00714-0 (2003). 13129865

[b15] Kanda S., Uemura A., Sakamoto Y. & Kita H. Vitrectomy with internal limiting membrane peeling for macular retinoschisis and retinal detachment without macular hole in highly myopic eyes. American journal of ophthalmology 136, 177–180 (2003). 12834688 10.1016/s0002-9394(03)00243-5

[b16] Nadal J., Verdaguer P. & Canut M. I. Treatment of retinal detachment secondary to macular hole in high myopia: vitrectomy with dissection of the inner limiting membrane to the edge of the staphyloma and long-term tamponade. Retina 32, 1525–1530, doi: 10.1097/IAE.0b013e3182411cb8 (2012). 22466478

[b17] Ikuno Y. . Vitrectomy and internal limiting membrane peeling for myopic foveoschisis. American journal of ophthalmology 137, 719–724, doi: 10.1016/j.ajo.2003.10.019 (2004). 15059711

[b18] Kim K. S., Lee S. B. & Lee W. K. Vitrectomy and internal limiting membrane peeling with and without gas tamponade for myopic foveoschisis. American journal of ophthalmology 153, 320–326 e321, doi: 10.1016/j.ajo.2011.07.007 (2012). 21982957

[b19] Kwok A. K., Lai T. Y. & Yip W. W. Vitrectomy and gas tamponade without internal limiting membrane peeling for myopic foveoschisis. The British journal of ophthalmology 89, 1180–1183, doi: 10.1136/bjo.2005.069427 (2005). 16113377 PMC1772841

[b20] Chuang L. H. . Macular hole repair by vitrectomy and internal limiting membrane peeling in highly myopic eyes. Retina 34, 2021–2027, doi: 10.1097/IAE.0000000000000183 (2014). 24859476

[b21] Taniuchi S., Hirakata A., Itoh Y., Hirota K. & Inoue M. Vitrectomy with or without internal limiting membrane peeling for each stage of myopic traction maculopathy. Retina 33, 2018–2025, doi: 10.1097/IAE.0b013e3182a4892b (2013). 23975004

[b22] Parolini B. . Indications and Results of a New L-Shaped Macular Buckle to Support a Posterior Staphyloma in High Myopia. Retina 35, 2469–2482, doi: 10.1097/IAE.0000000000000613 (2015). 26079474

[b23] Qi Y., Duan A. L., You Q. S., Jonas J. B. & Wang N. Posterior scleral reinforcement and vitrectomy for myopic foveoschisis in extreme myopia. Retina 35, 351–357, doi: 10.1097/IAE.0000000000000313 (2015). 25111687

[b24] Fujikawa M. . Scleral imbrication combined with vitrectomy and gas tamponade for refractory macular hole retinal detachment associated with high myopia. Retina 34, 2451–2457, doi: 10.1097/IAE.0000000000000246 (2014). 25062437

[b25] Lam R. F. . Pars plana vitrectomy and perfluoropropane (C3F8) tamponade for retinal detachment due to myopic macular hole: a prognostic factor analysis. American journal of ophthalmology 142, 938–944, doi: 10.1016/j.ajo.2006.07.056 (2006). 17157579

[b26] Li X., Wang W., Tang S. & Zhao J. Gas injection versus vitrectomy with gas for treating retinal detachment owing to macular hole in high myopes. Ophthalmology 116, 1182–1187 e1181, doi: 10.1016/j.ophtha.2009.01.003 (2009). 19375168

[b27] Wei Y. . Efficacy of vitrectomy with triamcinolone assistance versus internal limiting membrane peeling for highly myopic macular hole retinal detachment. Retina 33, 1151–1157, doi: 10.1097/IAE.0b013e31827b6422 (2013). 23508079

[b28] Lim L. S. . Prognostic factor analysis of vitrectomy for retinal detachment associated with myopic macular holes. Ophthalmology 121, 305–310, doi: 10.1016/j.ophtha.2013.08.033 (2014). 24139155

[b29] Ikuno Y. . Foveal anatomical status and surgical results in vitrectomy for myopic foveoschisis. Japanese journal of ophthalmology 52, 269–276, doi: 10.1007/s10384-008-0544-8 (2008). 18773264

[b30] Ortisi E., Avitabile T. & Bonfiglio V. Surgical management of retinal detachment because of macular hole in highly myopic eyes. Retina 32, 1704–1718, doi: 10.1097/IAE.0b013e31826b671c (2012). 23007668

[b31] Zhu S. Q. . The efficacy and safety of posterior scleral reinforcement using genipin cross-linked sclera for macular detachment and retinoschisis in highly myopic eyes. Br J Ophthalmol, doi: 10.1136/bjophthalmol-2015-308087 (2016). 26917677

[b32] Siam A. L., El Maamoun T. A. & Ali M. H. Macular buckling for myopic macular hole retinal detachment: a new approach. Retina 32, 748–753, doi: 10.1097/IAE.0b013e3182252a75 (2012). 21857392

[b33] Avila M. Y. & Navia J. L. Effect of genipin collagen crosslinking on porcine corneas. Journal of cataract and refractive surgery 36, 659–664, doi: 10.1016/j.jcrs.2009.11.003 (2010). 20362860

[b34] Jorge-Herrero E. . Biocompatibility and calcification of bovine pericardium employed for the construction of cardiac bioprostheses treated with different chemical crosslink methods. Artif Organs 34, E168–176, doi: 10.1111/j.1525-1594.2009.00978.x (2010). 20633147

[b35] Liu T. X., Luo X., Gu Y. W., Yang B. & Wang Z. Correlation of discoloration and biomechanical properties in porcine sclera induced by genipin. International journal of ophthalmology 7, 621–625, doi: 10.3980/j.issn.2222-3959.2014.04.06 (2014). 25161931 PMC4137195

[b36] Liu T. X. & Wang Z. Collagen crosslinking of porcine sclera using genipin. Acta ophthalmologica 91, e253–257, doi: 10.1111/aos.12172 (2013). 23710671

[b37] Wollensak G. & Spoerl E. Collagen crosslinking of human and porcine sclera. Journal of cataract and refractive surgery 30, 689–695, doi: 10.1016/j.jcrs.2003.11.032 (2004). 15050269

[b38] Xue A. . Posterior scleral reinforcement on progressive high myopic young patients. Optom Vis Sci 91, 412–418, doi: 10.1097/OPX.0000000000000201 (2014). 24509544

[b39] Saka N. . Long-term changes in axial length in adult eyes with pathologic myopia. Am J Ophthalmol 150, 562–568 e561, doi: 10.1016/j.ajo.2010.05.009 (2010). 20688315

[b40] Ichibe M. . Surgical management of retinal detachment associated with myopic macular hole: anatomic and functional status of the macula. American journal of ophthalmology 136, 277–284 (2003). 12888050 10.1016/s0002-9394(03)00186-7

[b41] Ando F., Ohba N., Touura K. & Hirose H. Anatomical and visual outcomes after episcleral macular buckling compared with those after pars plana vitrectomy for retinal detachment caused by macular hole in highly myopic eyes. Retina 27, 37–44, doi: 10.1097/01.iae.0000256660.48993.9e (2007). 17218913

[b42] Zhu Z., Ji X., Zhang J. & Ke G. Posterior scleral reinforcement in the treatment of macular retinoschisis in highly myopic patients. Clin Exp Ophthalmol 37, 660–663, doi: 10.1111/j.1442-9071.2009.02111.x (2009). 19788661

[b43] Ripandelli G. . Evaluation of primary surgical procedures for retinal detachment with macular hole in highly myopic eyes: a comparison [corrected] of vitrectomy versus posterior episcleral buckling surgery. Ophthalmology 108, 2258–2264, discussion 2265 (2001). 11733267 10.1016/s0161-6420(01)00861-2

[b44] O’Driscoll A. M., Goble R. R. & Kirkby G. R. Vitrectomy for retinal detachments with both peripheral retinal breaks and macular holes. An assessment of outcome and the status of the macular hole. Retina 21, 221–225 (2001). 11421010 10.1097/00006982-200106000-00004

[b45] Margheria R. R. & Schepens C. L. Macular breaks. 1. Diagnosis, etiology, and observations. American journal of ophthalmology 74, 219–232 (1972). 4559896

[b46] Gonvers M. & Machemer R. A new approach to treating retinal detachment with macular hole. American journal of ophthalmology 94, 468–472 (1982). 7137271 10.1016/0002-9394(82)90240-9

[b47] Ikuno Y. . Optical coherence tomographic findings of macular holes and retinal detachment after vitrectomy in highly myopic eyes. American journal of ophthalmology 136, 477–481 (2003). 12967801 10.1016/s0002-9394(03)00269-1

[b48] Bures-Jelstrup A. . Visual and anatomical outcome after macular buckling for macular hole with associated foveoschisis in highly myopic eyes. Br J Ophthalmol 98, 104–109, doi: 10.1136/bjophthalmol-2013-304016 (2014). 24169656

[b49] Mateo C., Bures-Jelstrup A., Navarro R. & Corcostegui B. Macular buckling for eyes with myopic foveoschisis secondary to posterior staphyloma. Retina 32, 1121–1128, doi: 10.1097/IAE.0b013e31822e5c32 (2012). 22027863

